# Phenylalanine-arginine β-naphthylamide could enhance neomycin-sensitivity on *Riemerella anatipestifer in vitro* and *in vivo*

**DOI:** 10.3389/fmicb.2022.985789

**Published:** 2023-01-11

**Authors:** Shiqi Liu, Junfa Liu, Ning Fu, Bunlue Kornmatitsuk, Zhuanqiang Yan, Junrong Luo

**Affiliations:** ^1^Jiangxi Provincial Key Laboratory for Animal Health, Institute of Animal Population Health, College of Animal Science and Technology, Jiangxi Agricultural University, Nanchang, China; ^2^Jinzhai County Agriculture and Rural Bureau, Jinzhai, Anhui, China; ^3^Wen's Group Academy, Xinxing, Guangdong, China; ^4^Chifeng Institute of Agricultural Sciences, Chifeng, China; ^5^Department of Clinical Sciences and Public Health, Faculty of Veterinary Science, Mahidol University, Nakhon Pathom, Thailand

**Keywords:** *Riemerella anatipestifer*, duck, antibiotic, phenylalanine-arginine β-naphthylamide, neomycin, efflux pump

## Abstract

*Riemerella anatipestifer* is an important duck pathogen responsible for septicemia and infectious serositis, which has caused great economic losses to the duck industry. Phenylalanine-arginine β-naphthylamide (PAβN) is an efflux pump inhibitor, which mainly reduces the efflux effect by competing with antibiotics for efflux pump channels. Here, we found that *R. anatipestifer* strain GD2019 showed resistances to gentamicin, amikacin, kanamycin, and neomycin. Notably, PAβN could significantly reduce the Minimal inhibitory concentrations (MICs) of neomycin on the GD2019 strain. Moreover, PAβN combined with neomycin significantly decreased bacterial loads, relieved pathological injury and increase survival rate (*p* < 0.05) for the ducks lethally challenged by the GD2019 strain. Therefore, our results suggested, *in vitro* and *in vivo,* PAβN could reduce neomycin-resistant of *R. anatipestifer.* Importantly, finding of this study provide a new approach for treating antibiotic-resistant *R. anatipestifer* infection.

## Introduction

*Riemerella anatipestifer* (*R. anatipestifer*), causing duck infectious serositis, is Gram-negative, short rod-shaped with non-spore-forming, non-motile bacterium, which belongs to the family *Flavobacteriaceae* (Kiss et al., 2007). *Riemerella anatipestifer* can be classified into at least 21 serotypes (Pathanasophon et al., 1995); it causes fibrinous pericarditis, perihepatitis, and airsacculitis in ducks of 1–8 weeks, leading to growth inhibition or death (Yang et al., 2020). Since *R. anatipestifer* was isolated and identified in ducks in 1932 (Hendrickson and Hilbert, 1932), it has spreaded rapidly to other regions of the world (Yuan et al., 2013), leading substantial economic losses to the duck industry.

Currently, clinical prevention and control of *R. anatipestifer* infection in ducks are mainly dependent on antibiotics and the most commonly used antibiotics are amides, cephalosporin, and aminoglycoside. However, recent studies showed that the isolates of *R. anatipestifer* were resistant to multi-antibiotics (Tzora et al., 2021). Antibiotic efflux is an important antibiotic-avoidance mechanism of bacteria, *via* recognizing and transporting out drugs (Li et al., 2020). It has been reported that the putative ATP-binding cassette superfamily (ABC) and resistance-nodulation-cell division (RND) efflux pump system plays a key role in antibiotic resistance of *R. anatipestifer* (Xin et al., 2017; Li et al., 2020; Wang et al., 2020). Phenylalanine-arginine β-naphthylamide (PAβN) is a protonophore inhibitor, which could compete for binding site with drugs in the process of transport (Opperman and Nguyen, 2015). PAβN shows effective inhibition of RND efflux pump in *Pseudomonas aeruginosa* and can be used as an adjuvant therapy (Renau et al., 1999, 2001, 2002, 2003; Lomovskaya et al., 2001; Watkins et al., 2003). Additionally, there are very few data on usage of efflux pump inhibitors combined with antibiotics for treatment of antibiotic-resistant *R. anatipestifer* infection in animal models (Opperman and Nguyen, 2015). In this study, the efflux pump inhibitor PAβN combined with neomycin was employed to treat the Muscovy ducks (*Cairina moschata*) infected with *R. anatipestifer*. It may also provide a theoretical guidance for the clinical use of efflux pump inhibitors for treatment of other bacterial infections in livestock.

## Materials and methods

### Bacterial strain and experimental ducks

*Riemerella anatipestifer* strain GD2019 was isolated from ducks with infectious serositis in Guangdong Province, China in 2019, and it was identified as serotype 2. The Tryptic Soy Broth (TSB) medium and Tryptic Soy Agar (TSA) medium were purchased from Becton, Dickinson, and Company (United States). The TSB-FBS or TSA-FBS medium for *R. anatipestifer* propagation TSB or TSA supplemented with 5% fetal bovine serum (FBS; BOVOGEN, Australia).

One-day-old healthy Muscovy ducks (*Cairina moschata*) were procured from Wen’s Foodstuffs Group Co., Ltd. (Guangdong, China). All ducks were maintained in our animal facility with 203 M duck feed (Wen’s Foodstuffs Group Co., Ltd., China). Feed and water were provided *ad libitum* during the experimentation process.

### Determination of antibiotic resistance of *Riemerella anatipestifer* strain GD2019

To determine antibiotic resistance of *R. anatipestifer* strain GD2019, the Kirby–Bauer disk diffusion method was performed as previously described with some modifications (Gao et al., 2018). Briefly, *R. anatipestifer* strain GD2019 was spread on TSA-FBS medium (10^9^ CFU/ml), antibiotics commonly used for treatment of *R. anatipestifer* infection in veterinary clinic, were placed on the medium. After 24 h, the inhibitory rings were observed and measured with the ruler. The gentamicin (12.5 μg/disk), amikacin (20 μg/disk), kanamycin (30 μg/disk), neomycin (20 μg/disk), enrofloxacin (5 μg/disk), ciprofloxacin (5 μg/disk), streptomycin (10 μg/disk), cotrimoxazole (23.75 μg/disk), tetracycline (30 μg/disk), doxycycline (30 μg/disk), ceftriaxone (30 μg/disk), ampicillin (10 μg/disk), amoxicillin (20 μg/disk), tylosintartrate (150 μg/disk), erythromycin thiocyanate (15 μg/disk), colistin sulfate (10 μg/disk), florfenicol (30 μg/disk), and blank disk were applied (Hangzhou Binhe Microorganism Reagent Co., Ltd., Hangzhou, China).

### Minimal inhibitory concentrations assay

To ensure PAβN could reduce antibiotics resistance on *R. anatipestifer* strain GD2019 *in vitro*, MICs assay were performed in the presence and absence of PAβN as previously described with some modifications (Chen et al., 2018). Briefly, *R. anatipestifer* strain GD2019 was seeded in TSB-FBS medium, shaken at 220 revolutions per minute (r/min) for 12 h. Add 100 μl of 10^5^ CFU/ml of microbial suspension to each well in a 96-well microtiter plate. Antibiotics (neomycin, kanamycin, gentamicin, and amikacin; Sigma, United States) were correspondingly added to the plate with serial 2-fold dilution from 128 to 0.25 μg/ml (1st–10th columns), then, PAβN (MedChemExpress Company, United States) dissolved in 0.05% DMSO (diluted with TSB) was added to the plate to make its concentration reached 40 μg/ml (Chen et al., 2018). Bacterial liquid and PAβN were added in 11 columns of each row, in the 12th column, only bacterial liquid was added, and the last two columns were used as control. The plate was then incubated at 37°C with 5% CO_2_. After 24 h, the OD_600nm_ values were measured and recorded immediately using Spectra-Max M2 (Molecular Devices, United States). The MIC was recognized as the lowest concentration of the antibiotics that can inhibit the visible growth of bacteria according to the Clinical and Laboratory Standards Institute’s (CLSI) 2-fold serial broth microdilution method (CLSI, 2017), a reduction in MIC of at least four-fold was considered as indicative of efflux (Xian-Zhi et al., 2016).

### Safety evaluation of PAβN inhibitory doses and enhancing effects of PAβN on neomycin against *Riemerella anatipestifer* in ducks

The animal study was approved by the Institutional Animal Care and Use Committee of Jiangxi Agricultural University (Jiangxi, China) and animals were treated in accordance with the regulations and guidelines of this committee. The toxicity evaluation of PAβN and the enhancing effects of PAβN on neomycin against *R. anatipestifer* infection *in vivo* were performed as previously described with some modifications (Xu et al., 2020; Yang et al., 2020). Briefly, for the toxicity assessment, 20 5-day-old Muscovy ducks were randomly divided into four groups (five ducks per group) and were housed in four separate rooms. On day one, ducks in group 1 were intramuscularly injected with 0.5 ml of 0.05% DMSO and served as controls. Ducks in group 2, 3, and 4 were intramuscularly injected with 0.5 ml of 0.05% DMSO containing PAβN at a dose of 10, 20, and 40 μg/g of body weight (BW), respectively. All groups were treated for 3 days. Ducks death were observed and recorded until the trial ended. In addition, the body weight of each duck was measured every 4 days, and all ducks were necropsied at 28 days post inoculation (d.p.i.), blood samples were collected for blood cells and blood biochemical tests.

For the PAβN enhanced neomycin against *R. anatipestifer* experiment, 40 14-day-old Muscovy ducks (Specific antibodies negative to *R. anatipestifer*) were randomly divided into four groups (10 ducks per group) and were housed in four separate rooms. Ducks in group 1 were first intramuscularly injected with 0.5 ml of TSB-FBS and served as controls. Ducks in group 2, 3, and 4 were first intramuscularly injected with 0.5 ml of TSB-FBS containing minimum lethal dose (5 × 10^5^ CFU) of the GD2019 strain. After challenging, ducks in group 1 were intramuscularly injected with 0.5 ml of 0.05% DMSO, ducks in group 2 were 0.5 ml of 0.05% DMSO containing PAβN at a dose of 40 μg/g of BW, ducks in group 3 were 0.5 ml of 0.05% DMSO containing neomycin at a dose of 8 μg/g of BW, ducks in group 4 were 0.5 ml of 0.05% DMSO containing neomycin at a dose of 8 μg/g of BW, and PAβN at a dose of 40 μg/g of BW. These treatments lasted for 3 days. The death of duck was observed and recorded for the next 7 days.

### Recovery of bacteria from organs

Twenty 15-day-old Muscovy ducks were divided into four groups (five ducks per group) and were housed in four separate rooms. Ducks in group 1 were intramuscularly injected with TSB-FBS medium and served as controls. Ducks in group 2, 3, and 4 were intramuscularly injected with a sublethal dose (0.5 × 10^3^ CFU) of the GD2019 strain. After challenging, ducks were treated with the same drugs as described above for 3 days. All ducks were sacrificed after treatment, the heart, liver, and brain were homogenized in sterile 1 × PBS, and the number of bacteria was determined by CFU on the TSA-FBS medium as previously described with some modifications (Wang et al., 2008). In addition, the tissues of heart, liver, and brain were collected and examined by histopathology.

## Results

### Antibiotic resistance test results

**Figure 1:**
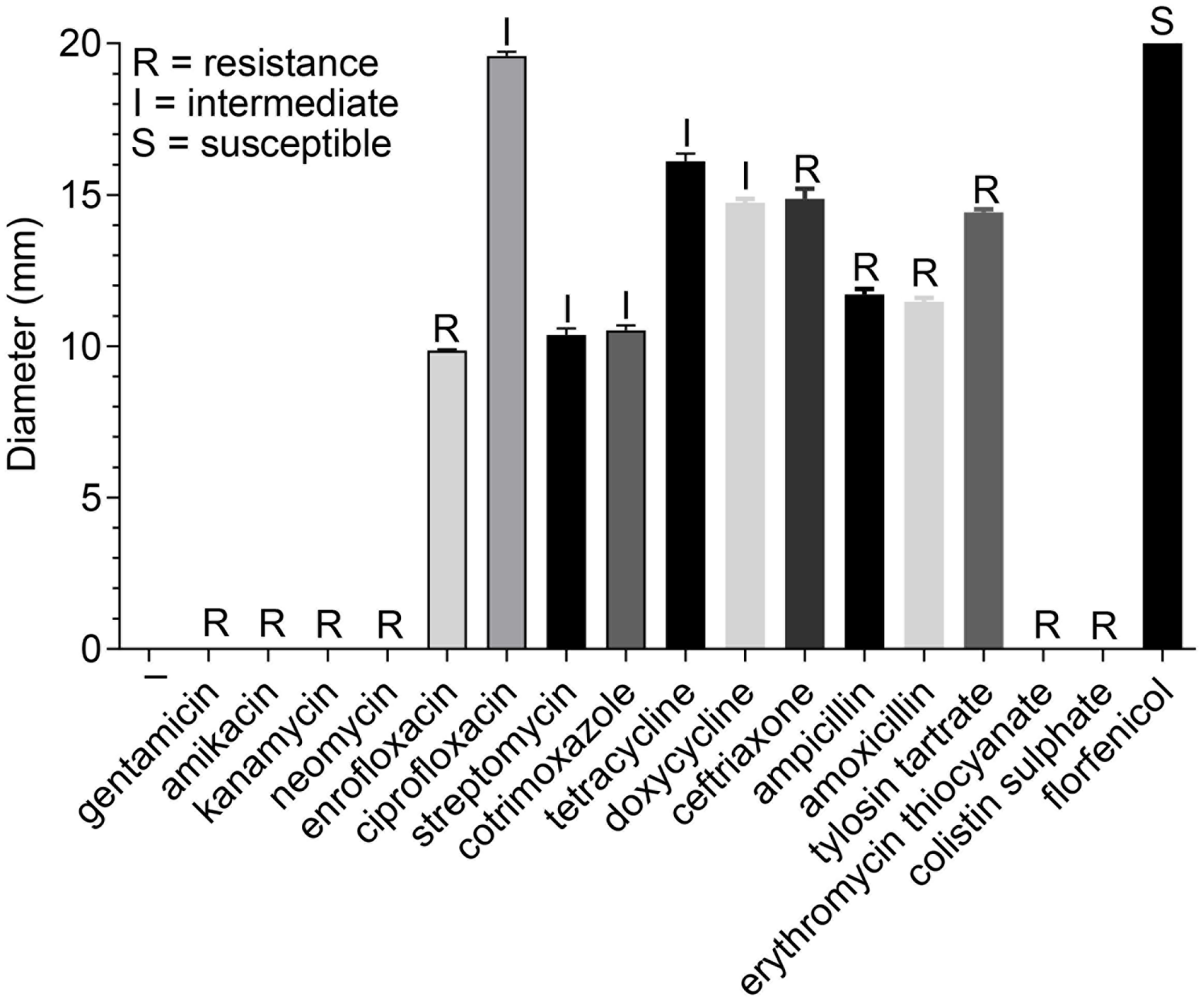
Determination of antibiotic resistance of *Riemerella anatipestifer* strain GD2019.

To screen for effective antibiotics, we performed the antimicrobial susceptibility test to assess the antibiotic resistance of the GD2019 strain. As shown in Figure 1, the GD2019 strain showed resistances to aminoglycosides, fluoroquinolones, chloramphenicol, etc., indicating that the GD2019 strain is multi-drug resistant. In the drug resistance test, we found that neomycin, kanamycin, gentamicin, and amikacin were all aminoglycosides.

### MICs assay results

**Figure 2:**
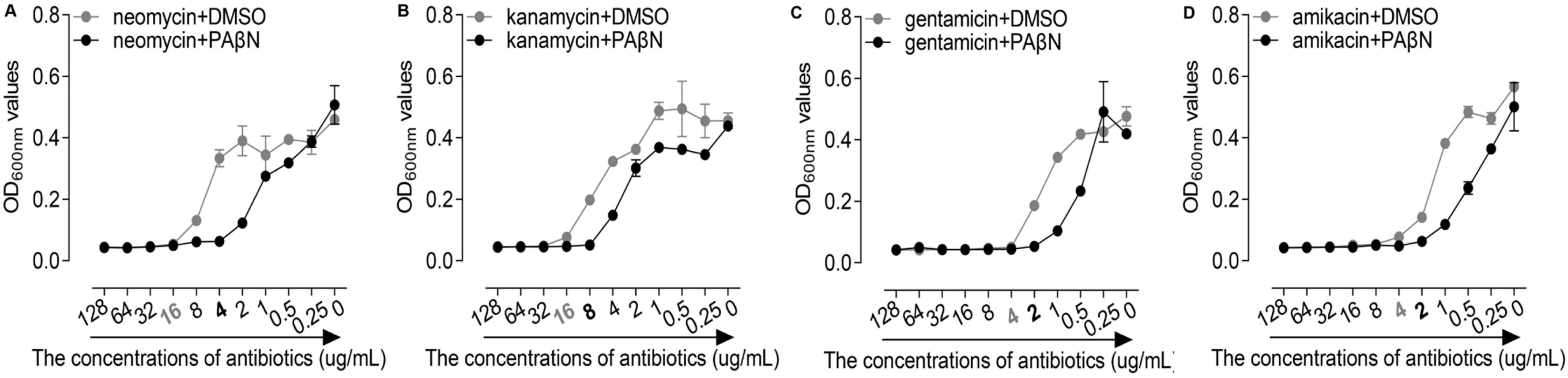
Effects of phenylalanine-arginine β-naphthylamide (PAβN) combined with antibiotics against *Riemerella anatipestifer* strain GD2019 by MIC method. **(A)** Neomycin, **(B)** Kanamycin, **(C)** Gentamicin, and **(D)** Amikacin (mean ± SD, *n* = 3).

In this study, we performed MICs assay to assess the antibiotics against the GD2019 strain in the presence and absence of PAβN. As a result, PAβN significantly reduced the MIC of neomycin in the GD2019 strain (Figure 2A), indicating that PAβN can enhance the antimicrobial activity of neomycin against the GD2019 stain *in vitro*. Interestingly, PAβN could not significantly enhance the antimicrobial activity of kanamycin, gentamicin, and amikacin (Figures 2B–D); indicating that the GD2019 strain had multiple mechanisms to resist antibiotics.

### PAβN safety assay

**Figure 3:**
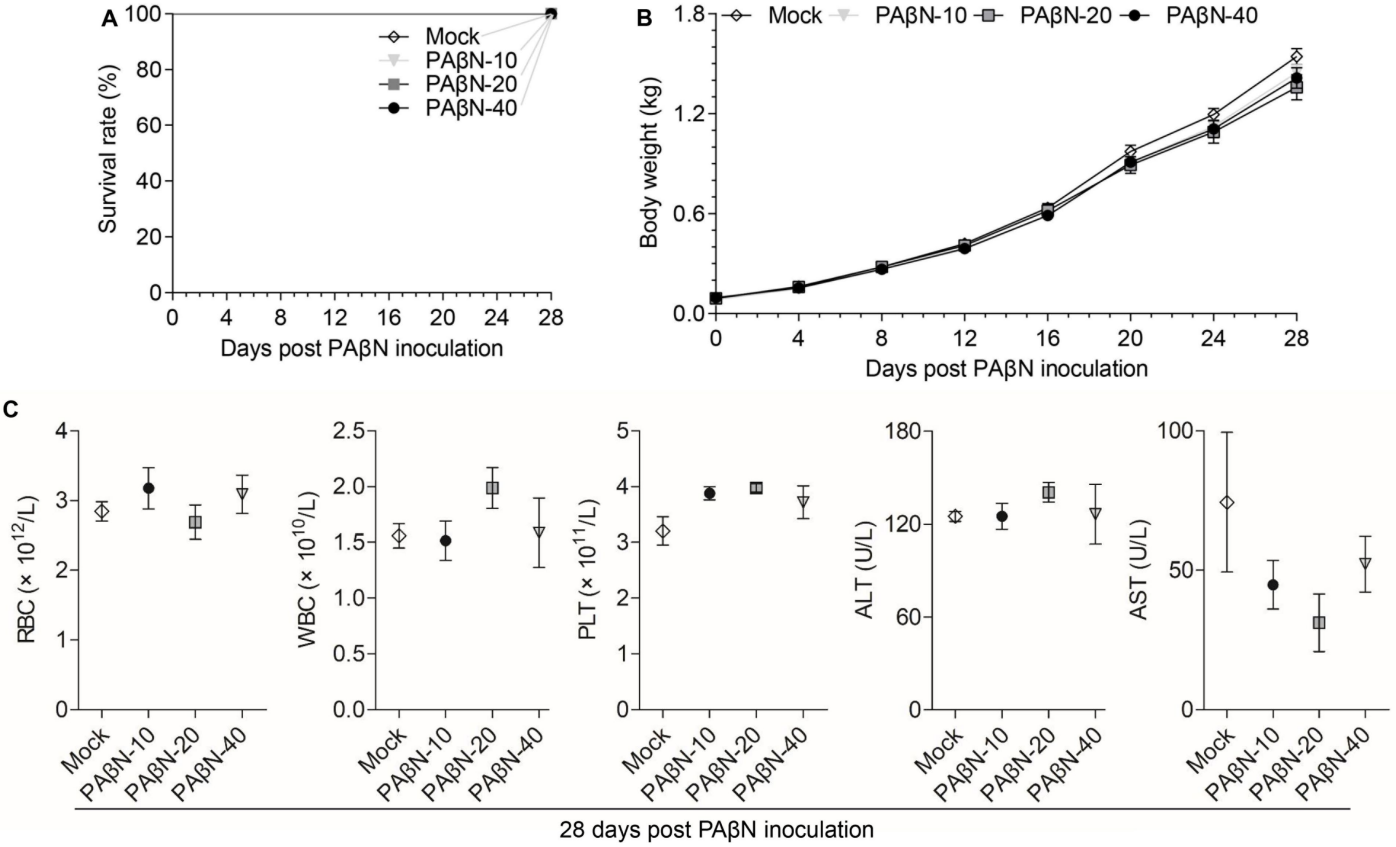
Safety evaluation of PAβN *in vivo*. **(A)** Survival curves of ducks injected with PAβN. **(B)** Body weights of ducks injected with PAβN. **(C)** Results of blood cells and blood biochemical tests (mean ± SD, *n* = 5). RBC, red blood cell; WBC, white blood cell; PLT, platelet; ALT, alanine aminotransferase; and AST, aspartate aminotransferase.

In this study, the toxicity study of PAβN *in vivo* was studied *via* intramuscular injection and the deaths, body weight changes, blood cell counts, and AST and ALT detection of PAβN-inoculated ducks were recorded and analyzed (Figure 3).

### Result of enhancing effects of PAβN on neomycin against *Riemerella anatipestifer* in ducks

**Figure 4:**
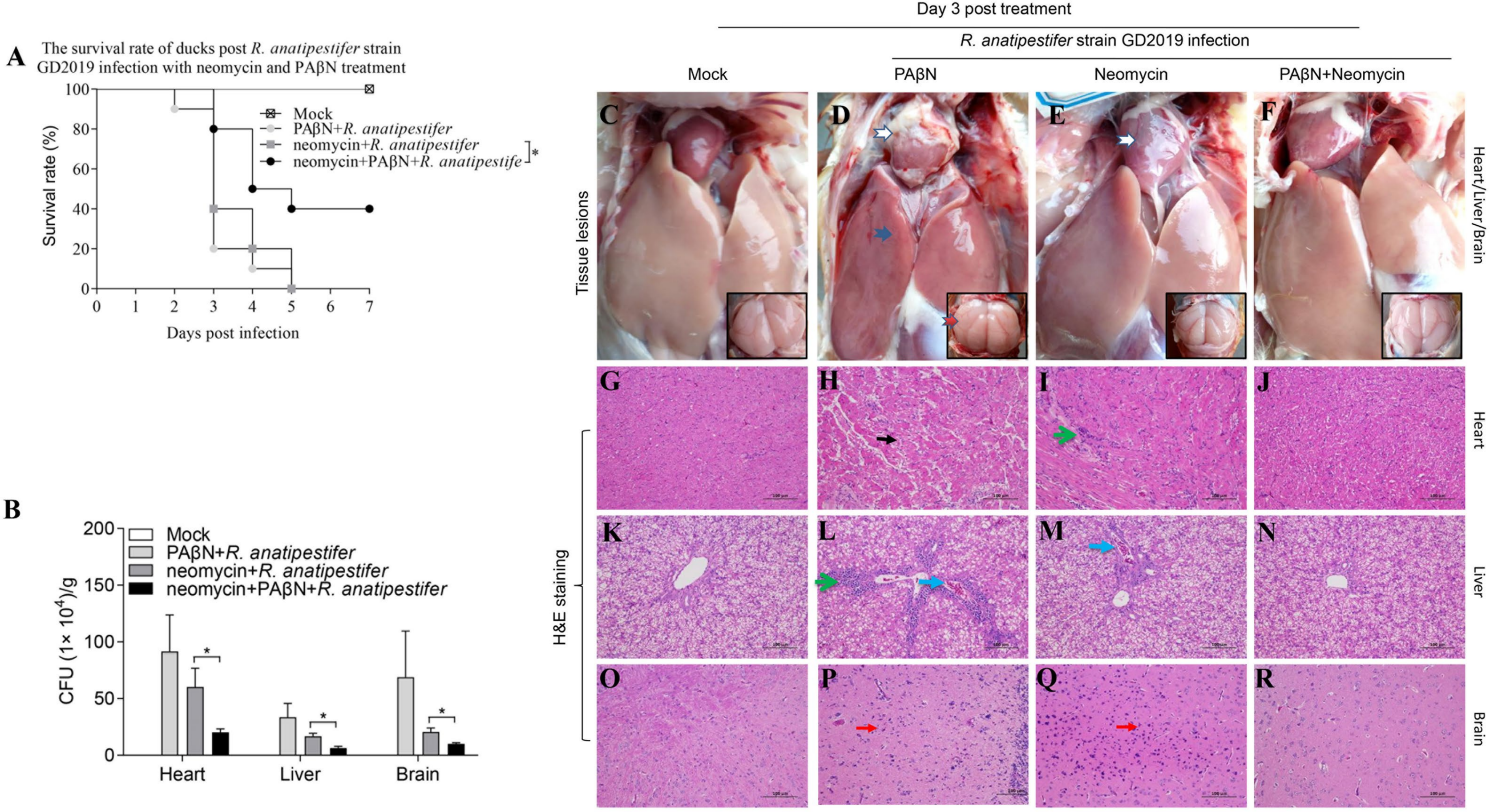
Enhancing effect of PAβN for neomycin against GD2019 infection in ducks. **(A)** The survival rate of ducks (*n* = 10). **(B)** Bacterial loads in heart, liver, and brain (mean ± SD, *n* = 5. ^*^stands for *p* < 0.05). **(C–F)** Macroscopic pictures of heart, liver, and brain. **(G–R)** H&E-stained heart, liver, and brain tissue section. →Pericarditis, →Perihepatitis, →Meningitis, →Myocardial necrosis, →Inflammatory cell infiltration, →Congestion, →Microgliosis.

The results provided a reference for the proper use of PAβN in ducks, 14-day-old Muscovy ducks were intramuscularly injected with minimum lethal dose (5 × 10^5^ CFU) of the GD2019 strain, then treated with PAβN + neomycin for 3 days. We found that PAβN + neomycin treatment could significantly reduce bacterial loads and pathological changes in heart, liver, and brain and increase survival rate (*p* < 0.05) from challenge with the GD2019 strain (Figure 4), indicating that PAβN can be used as one of choice for prevention and control neomycin-resistant *R. anatipestifer* in duck farms.

## Discussion

In the present study, we reported that PAβN, an efflux pump inhibitor reduced neomycin resistance of the GD2019 isolate, a multi-drug resistant strain *in vitro* and enhanced neomycin-sensitivity against the GD2019 strain infection in ducks, which might help to control *R. anatipestifer* in duck farms and provide a certain reference value to use of external drainage pump inhibitors to enhance a disease treatment in other livestock.

Collectively, the present results confirmed that *R. anatipestifer* causes severe fibrinous pericarditis, perihepatitis and fatal in ducks. We infected 14-day-old ducks with the GD2019 strain at different doses *via* intramuscular injection, the typical symptoms such as fibrinous pericarditis, perihepatitis were successfully reproduced and the LD_50_ of the GD2019 strain is 5 × 10^3^ CFU, these results strongly suggested that *R. anatipestifer* posed a major threat in duck production worldwide.

In recent years, bacterial resistance has attracted widespread attention, and more scholars have sought to solve the problem. Our present data confirmed that the GD2019 strain showed resistant to multiple antibiotics, indicating that the *R. anatipestifer* prevalent strains may exhibited multiple drug resistance in Guangdong, China. Unfortunately, it was reported that *R. anatipestifer* vaccines cannot provide cross-protection between serotypes (Yang et al., 2020); antibiotics are still the first choice for the prevention and control the infection of *R. anatipestifer* in duck farms.

Antibiotic resistant bacteria are mainly mediated by modifying the antibiotic target, inactivating the antibiotic by hydrolysis, and minimize the intracellular concentrations of the antibiotic by antibiotic efflux mechanism (Wright, 2011). Overexpressed RND efflux pumps are major components in the development of the multidrug resistance phenotype in Gram-negative bacteria, which actively pump biocides and antibacterial agents from the periplasm to outside cells (Opperman and Nguyen, 2015). However, at present, RND efflux pumps are poorly understood to *R. anatipestifer*, and only one putative RND transporter was identified preliminarily, which contributes to the export of some drugs belonging to aminoglycoside and detergent (Xin et al., 2017).

It has been proved (Wang et al., 2020) that RND efflux pump protein is a conserved protein in *R. anatipestifer*, which not only can excrete aminoglycosides but also may be implicated them in diverse phenotypes including metabolism, biofilm production, iron acquisition, fitness, and virulence (Laura, 2006; Caughlan et al., 2012; Dinesh et al., 2013; Wang et al., 2020). PAβN was first reported in 1999 (Renau et al., 1999) and subsequently demonstrated as a broad-spectrum efflux pump inhibitor, which could significantly reduce fluoroquinolone resistance in *Pseudomonas aeruginosa* (Lomovskaya et al., 2001). In this study, PAβN significantly reduced MIC value of neomycin in the GD2019 strain *in vitro*, indicating that the GD2019 strain was resistant to neomycin through the efflux pump, actually most of the genes encoding these multidrug resistance pumps are normal constituents of bacterial chromosomes, it indicates that some of these genes have a relatively high level of constitutive expression and confer so-called intrinsic resistance to antibiotics (Lomovskaya et al., 2001). Therefore, it could be proposed that high expression of the RND efflux pump in the GD2019 strain might involve in aminoglycoside resistance. Additionally, PAβN could reduce the drug resistance through competitive efflux channels with aminoglycosides (Opperman and Nguyen, 2015), but PAβN could not reduce the resistance of other antibiotics, indicating that the GD2019 strain has multiple mechanisms of drug resistance.

Phenylalanine-arginine β-naphthylamide, as an inhibitor of efflux pump, has not been reported for disease treatment *in vivo*. For a reagent/drug used in the treatment, safety is the top concern. Acute and subacute toxicity are two important indexes for reagent/drug safety evaluation (Vakili et al., 2017), which can help to determine the scope and the length of time, and to reduce side effects. In this study, we intramuscularly injected PAβN to ducks and determined a relative safe dose of 40 μg/g of BW by recording the death, body weight changes, blood cell counts, and AST and ALT of PAβN-treated ducks, which provided a reference for the proper use of PAβN in ducks. However, the protection rate did not reach 100%.

Furthermore, it has been documented that dithiazolethione derivatives (DTT10) were identified as an effective inhibitor for the efflux pump major facilitator superfamily (MFS) of the *Staphylococcus aureus*, higher concentrations of DTT10 can inhibit the efflux aisle by competing with MFS efflux substrates, while lower concentrations of DTT10 can also inhibit MFS efflux ciprofloxacin. Additionally, DTT10 could also reduce bacterial load in muscle and skin tissue in a zebrafish model of *Staphylococcus aureus* infection (Lowrence et al., 2016). Similar to the present results, the combination of efflux pump inhibitors and antibiotics can improve the treatment effect. Hong et al. (2021) also demonstrated that thioimidazine has an inhibitory effect on the efflux pump MFS of *Staphylococcus aureus*. Through molecular docking and molecular dynamics simulations, they observed that thioimidazine blocked the substrate binding to MFS, reducing its activity, which also demonstrated synergistic anti-staphylococcal activity *in vitro* with losacillin, thioimidazine, and tetracycline. *In vivo* pharmacological inhibition experiment showed that the combination of losacillin, thioimidazine, and tetracycline significantly reduced the number of bacterial colony-forming units in the viscera of mice infected with *Staphylococcus aureus* peritonitis, and the treatment alleviated the primary inflammatory pathology. Therefore, Hong et al. (2021) suggested that the combination of efflux pump inhibitors and antibiotics is a new anti-staphylococcal and anti-inflammatory strategy, which provides well-response antibacterial activity and significant inhibitory effects on inflammation. Moreover the efflux pump inhibitors (trimethoprim and sertraline) combined with levofloxacin were also used to treat *G. mellonella* larvae infected with *Pseudomonas aeruginosa* with high expression of the efflux pump MexAB-OprM gene, compared with levofloxacin monotherapy, a better therapeutic effect was produced (Dougal et al., 2015). These results are similar to those of our clinical trial, demonstrating enhancing effect of efflux pump inhibitors combined with antibiotics for treatment of bacterial diseases.

In our experiments, it was proved that the efflux pump inhibitor PAβN can reduce the resistance of *R. anatipestifer* to neomycin both *in vitro* and *in vivo*, and can enhance the bactericidal activity of conventional antibiotics. However, there are still several important questions remain for us to do. For instance, what is the exact potential mechanism of PAβN reducing the resistance of *R. anatipestifer* to neomycin? How to develop new efflux pump inhibitors? Can the combination of new efflux pump inhibitors and antibiotics reduce the resistance of other *R. anatipestifer*? Elucidation of these questions will help us to develop a new treatment regimen to control multi-drug resistant *R. anatipestifer* in ducks.

In conclusion, the efflux pump inhibitor PAβN could reduce neomycin-resistance of the GD2019 strain *in vitro*, and increase the survival rate of neomycin-treatment against *R. anatipestifer* infection, indicating that it might solve the problem of multi-drug resistance of *R. anatipestifer* and be used to control *R. anatipestifer* infection in duck farms.

### Histological staining

Histological staining was performed as previously described with some modifications (Yang et al., 2020). Briefly, tissue samples of heart, liver, and brain of the ducks from the experimental groups were fixed in 10% formalin for 36 h at room temperature, and then dehydrated in graded ethanol, embedded in paraffin, cut in 5-μm section, and mounted onto glass slides. After the sections were deparaffinized, rehydrated, and stained with hematoxylin and eosin (H&E), the slides were examined and analyzed with conventional light microscopy (Nikon, Japan).

### Statistical analysis

Statistical comparisons were performed using GraphPad Prism software 5.0 (GraphPad, San Diego, CA, United States) and the differences among the experimental groups (body weight, WBC, RBC, PLT, AST, ALT, survival rate, and CFU) were evaluated by the ANOVA and Mann–Whitney accordingly. *p* values <0.05 were considered statistically significant.

## Data availability statement

The original contributions presented in the study are included in the article/supplementary material; further inquiries can be directed to the corresponding author.

## Ethics statement

The animal study was reviewed and approved by Institutional Animal Care and Use Committee of Jiangxi Agricultural University.

## Author contributions

SL, JLu, and JLi conceived and designed the experiments. SL and JLi performed the experiments. SL analyzed and organized the data. JLi, ZY, NF, and JLu contributed reagents, materials, and analysis tools. SL, JLu, and BK wrote the paper. JLu and JLi checked and finalized the manuscript. All authors contributed to the article and approved the submitted version.

## Funding

This work was supported by the transverse project of Jiangxi Agricultural University (#2021JXAUHX036), the Guangdong Province Sailing Plan (#2069999), the Guangdong Province Science and Technology Innovation Strategy Special Fund (#2018B030315002), and the Research Fund of the Wen’s Foodstuffs Group Co., Ltd. (#ZB20180801CCG002). The funder was not involved in the study design, collection, analysis, interpretation of data, the writing of this article, or the decision to submit it for publication.

## Conflict of interest

JLi and ZY were employed by Wen's Group Academy, Guangdong, China.

The remaining authors declare that the research was conducted in the absence of any commercial or financial relationships that could be construed as a potential conflict of interest.

## Publisher’s note

All claims expressed in this article are solely those of the authors and do not necessarily represent those of their affiliated organizations, or those of the publisher, the editors and the reviewers. Any product that may be evaluated in this article, or claim that may be made by its manufacturer, is not guaranteed or endorsed by the publisher.
